# Racial differences in cardiovascular events and adverse pregnancy outcomes among pregnant individuals with SLE

**DOI:** 10.1136/lupus-2025-001846

**Published:** 2026-09-15

**Authors:** Rashmi Dhital, Rebecca J Baer, Dilli R Poudel, Tracy M Frech, Christina Chambers

**Affiliations:** 1 Department of Medicine, Division of Rheumatology and Immunology, Vanderbilt Health, Nashville, Tennessee, USA; 2 Department of Pediatrics, Division of Environmental Science and Health, University of California San Diego, La Jolla, California, USA; 3 The California Preterm Birth Initiative, University of California San Francisco, San Francisco, California, USA; 4 Herbert Wertheim School of Public Health and Human Longevity Science, University of California San Diego, La Jolla, California, USA

**Keywords:** Cardiovascular Diseases, Lupus Erythematosus, Systemic, Epidemiology

## Abstract

**Objective:**

We aimed to characterise differences in the occurrence of maternal cardiovascular events (CVEs) and adverse pregnancy outcomes (APOs) in each of four racial/ethnic groups compared with non-Hispanic white women with SLE and to evaluate whether the relationship between CVEs and APOs differed across or within racial/ethnic groups.

**Methods:**

Using a California population-based birth cohort (2005–2020), we identified pregnant individuals with SLE via International Classification of Diseases codes from maternal discharge records. Self-reported racial/ethnic groups were categorised as Asian, Hispanic, non-Hispanic black, non-Hispanic white and other. Individuals with reported race or ethnicity as ‘unknown’ or ‘not stated’ were excluded from the study. Log-linear regression estimated adjusted relative risks (aRRs) of CVEs and APOs in each racial/ethnic group compared with non-Hispanic white individuals, adjusting for a propensity score derived from maternal age, payer, education, comorbidities and SLE-related variables. We performed CVE-stratified analyses for racial/ethnic differences in APOs and examined associations between CVEs and APOs within each racial/ethnic group.

**Results:**

Among 8188 liveborn singletons from women with SLE, non-Hispanic black women had the highest prevalence of maternal CVEs (4.0% vs 1.6–2.0% in other groups), maternal APOs (24.6% vs 13.5–17.2%) and infant APOs (54.6% vs 34.6–49.4%). Relative to non-Hispanic white women, non-Hispanic black women had an aRR of 1.6 (95% CI 0.9 to 2.6) for maternal CVEs and 1.3 (95% CI 1.1 to 1.6) for maternal APOs, whereas infant APO risks were significantly higher across three of four racial/ethnic groups compared with non-Hispanic white women.

**Conclusions:**

Non-Hispanic black women exhibited the highest absolute burden for the adverse outcomes studied. Relative to non-Hispanic white individuals, elevated adjusted risks based on race/ethnicity were most consistent for infant APOs, whereas elevated risks for maternal APOs and CVEs were found primarily among non-Hispanic black individuals. Social drivers of health need consideration while caring for pregnant individuals with SLE.

**WHAT IS ALREADY KNOWN ON THIS TOPIC**

- Individuals with SLE face an increased risk of cardiovascular events (CVEs) during pregnancy as well as adverse pregnancy outcomes (APOs). Racial and ethnic differences in SLE outcomes are well recognised, but the interplay between race/ethnicity, maternal CVEs and APOs remains poorly understood.

**WHAT THIS STUDY ADDS**

- Non-Hispanic black individuals had the highest absolute burden of maternal CVEs, maternal APOs and infant APOs. Across racial/ethnic groups, maternal CVEs were associated with a higher risk of maternal APOs and to a lesser extent, infant APOs. Regardless of CVE status, non-Hispanic black individuals consistently experienced numerically higher rates of APOs than other racial/ethnic groups.

**HOW THIS STUDY MIGHT AFFECT RESEARCH, PRACTICE OR POLICY**

- Our findings help to individualise the risks of CVEs and APOs across racial/ethnic groups among individuals with SLE, highlighting the need to incorporate both cardiovascular risk assessment and social drivers of health into the care of pregnant individuals with SLE.
- By clarifying the influences of race/ethnicity and CVEs on APOs, this study informs the design and evaluation of cardio-obstetric care models within rheumatology to improve maternal and child health outcomes.

## Introduction

 Pregnant individuals with SLE have about a sixfold higher risk of maternal cardiovascular events (CVEs) during pregnancy and early postpartum compared with the general obstetric population. This risk approximately doubles in the presence of lupus nephritis (LN) and triples in SLE with antiphospholipid syndrome (APS).^1^ SLE is also associated with a 2–4-fold higher risk of adverse pregnancy outcomes (APOs) including pre-eclampsia, preterm birth (PTB) and small for gestational age (SGA) infants.^2 3^ The occurrence of CVEs during pregnancy is associated with increased risk of APOs, with LN and APS conferring incremental risk. For example, in a California birth cohort, pregnant individuals with SLE had more than twice the risk of PTB in pregnancies complicated by CVEs compared with those without CVEs (47.2% vs 20.8%).^4^ This pattern was even more pronounced among individuals with LN and APS, in whom PTB increased from 44.0% to 73.1% and from 30.0% to 52.5%, respectively, in the presence of CVEs.^4^

Significant racial and ethnic differences in SLE outcomes have been well described in the literature. Black and Hispanic individuals with SLE experience more severe disease manifestations and faster accumulation of organ damage compared with white individuals.^5–7^ Black and Hispanic individuals also experience higher risks of pregnancy complications including pre-eclampsia, caesarean delivery, PTB and fetal growth restriction.^8 9^ In a California-wide birth cohort, non-Hispanic black women comprised a greater proportion of SLE pregnancies with CVEs than those without CVEs (17.6% vs 9.1%).^4^ This aligns with national data showing that cardiovascular conditions disproportionately affect black women and contribute substantially to maternal mortality.^10^

Despite the recognised risks of both CVEs and APOs in SLE pregnancies, and the known influence of race and ethnicity on SLE outcomes, the interplay between race/ethnicity, maternal CVEs and APOs remains poorly understood. Clarifying the impact of CVEs on APOs across racial/ethnic groups could inform targeted cardio-obstetric care pathways for SLE pregnancies. Our aim was to use a large California-wide birth cohort of pregnant individuals with SLE to first characterise differences in the occurrence of maternal CVEs and maternal and infant APOs in each of four race/ethnic groups compared with non-Hispanic white women among pregnant individuals with SLE. Second, our aim was to evaluate whether the risk of APOs in the presence or absence of CVEs differed across the same four racial/ethnic groups compared with non-Hispanic white women. Finally, we sought to evaluate the extent to which CVEs were associated with risk of maternal or infant APOs within each of the five racial/ethnic groups.

## Methods

### Study design and data source

We conducted a retrospective population-based cohort study using the Study of Outcomes in Mothers and Infants (SOMI), a cohort of singleton liveborn infants in California, from the years 2005–2020. SOMI includes linked infant birth and death certificate data from California Vital Statistics to maternal and infant hospital discharge, emergency department and ambulatory surgery records from the California Department of Health Care Access and Information (HCAI), from 1 year before to 1 year after delivery.^11^

### Study population

Pregnant women with SLE were identified using the presence of any International Classification of Diseases, 9th Revision, Clinical Modification (ICD-9-CM: 710.0) or 10th Revision, Clinical Modification (ICD-10-CM: M32.1x, M32.8, M32.9) code on discharge records during pregnancy and/or birth admission.

### Exposure

The primary exposure of interest was maternal self-reported race/ethnicity, categorised as Asian, Hispanic, non-Hispanic black, non-Hispanic white and other (American Indian/Alaska Native, Native Hawaiian/Pacific Islander, other race, two or more races). Individuals with reported race or ethnicity as ‘unknown’ or ‘not stated’ were excluded from the study, as race/ethnicity was the primary exposure of interest. A secondary exposure was CVE status.

### Outcomes

The primary endpoints of interest were maternal CVEs and APOs. Maternal CVEs were identified using ICD codes for acute myocardial infarction, stroke, heart failure and peripartum cardiomyopathy, myocarditis, pericarditis, dysrhythmia and venous thromboembolism (pulmonary embolism or deep vein thrombosis) during pregnancy (online supplemental table 1).

APOs included maternal and infant APOs, identified during or up to 1 year after delivery admission. For this study, maternal APOs were defined as:

- Non-cardiac components of severe maternal morbidity (SMM) as defined by the Center for Disease Control and Prevention (CDC), identified using ICD codes in HCAI records for acute renal failure, acute respiratory distress syndrome, disseminated intravascular coagulation, amniotic fluid embolism, blood transfusion, severe anaesthesia complications, sepsis, hysterectomy, temporary tracheostomy and ventilation (online supplemental table 2).^12^
- Maternal intensive care unit (ICU) utilisation during delivery admission, ascertained from vital statistics records
- Maternal readmissions within 1 year postpartum, ascertained from HCAI records.
- Maternal deaths within the first year after delivery, ascertained from HCAI records when the hospital discharge record indicated discharge status=died and/or linked death records.

Infant APOs were identified from Vital Statistics and infant discharge records, and included

- PTB (delivery prior to 37 weeks of gestational age (WGA)), calculated from gestational age at birth from vital statistics records.
- SGA (birth weight<10 th percentile for gestational age and sex),^13^ calculated from variables in vital statistics records.
- ICU utilisation during the delivery admission, ascertained from vital statistics records.
- Infant readmission (readmission within 1 year of life), ascertained from HCAI records.
- Infant mortality (death within 1 year of life) ascertained from HCAI records when the hospital discharge record indicated discharge status=died and/or linked death records.

Spontaneous PTB was defined by the presence of indicators including preterm premature rupture of the membranes (PPROM), premature labour or tocolytic administration. Provider-initiated PTB were those without PPROM, premature labour or tocolytic administration who had a documentation of ‘medical induction’, ‘artificial rupture of membranes’ or caesarean delivery on birth certificate or hospital discharge records.

SMM is an index developed by the CDC and is defined as unexpected labour and delivery outcomes with serious short and long-term health implications for the mother. It includes cardiac components such as myocardial infarction, dysrhythmia, heart failure and stroke, as well as non-cardiac components.^12^

### Covariates

Covariates selected from maternal discharge records included those previously associated in the literature with risks of both CVEs and APOs, including maternal sociodemographic variables (age, payer, maternal education), prepregnancy body mass index (BMI), clinical comorbidities (pre-existing and gestational diabetes mellitus and hypertension, pregnancy-induced hypertension, hyperlipidaemia, depression and substance use during pregnancy) and SLE-related variables (concurrent LN or APS). Information on parity was obtained from birth certificates.

### Statistical analyses

We first compared baseline sociodemographic characteristics and clinical comorbidities between pregnant individuals with SLE across the five racial/ethnic groups using χ^2^ test. We then quantified the occurrence of CVEs and the incidence of composite and individual APOs by racial/ethnic category. These outcomes were summarised as counts and percentages for each of the five racial/ethnic groups, and crude relative risks (RR) were estimated for Asian, Hispanic, non-Hispanic black and other racial/ethnic groups compared with non-Hispanic white individuals as the reference group. Non-Hispanic white individuals were chosen as reference group, as prior studies have generally reported lower risks of adverse pregnancy as well as cardiovascular outcomes in this group.^4 8–10^

In our primary analyses, we used log-linear regression with a Poisson distribution to estimate the adjusted RRs (aRRs) and 95% CIs for CVEs and APOs among Asian, Hispanic and non-Hispanic black women, using non-Hispanic white women as the reference group, adjusting for race/ethnicity-specific propensity scores.

In secondary analyses, using the same statistical modelling approach, we conducted stratified analysis to explore whether association between race/ethnicity and APOs differed by CVE status. For this, we stratified pregnancies into those with and without a CVE, and within each stratum, modelled race/ethnicity as the exposure (with non-Hispanic white as the reference group) and any maternal APO and any infant APO as the outcomes, adjusting for corresponding race/ethnicity-specific propensity scores. Additionally, we examined the associations between CVE and risk of APOs within each racial/ethnic group, with CVE as the exposure and any maternal APO and any infant APO as outcomes, adjusting for the corresponding propensity score for CVE in each model. Last, we repeated the analyses restricting only to nulliparous women to evaluate the effect of parity.

We created race/ethnic group-specific propensity scores to summarise covariates rather than adjusting for them individually to avoid overfitting due to the infrequent number of outcomes in some racial/ethnic strata. To build the propensity score models, non-Hispanic white women served as the reference category and were modelled as a binary indicator (yes vs no). Separate logistic regression models were fitted with the covariates as predictors, yielding propensity scores that represented the predicted probability of belonging to that race/ethnic category, conditional on the covariates. Variables that could be continuous (age, BMI) were matched on variable groupings (age groups: <18 years, 18–34 years and >34 years; BMI groups: <18.5 kg/m^2^, 18.5 to <25 kg/m^2^, 25–30 kg/m^2^ and ≥30 kg/m^2^) to maximise matching opportunities. In the stratified analysis where CVE was the exposure, the propensity score was calculated using the same covariates and methods to represent the predicted probability of having a CVE.

An available-case approach was used for covariates included in the propensity score models. Individuals with the missing covariate data were retained in the propensity score estimation, and for such individuals, the corresponding indicator variable contributed a value of 0 in the propensity score model. Thus, propensity scores were estimated using the available covariate information for each individual. Most covariates included in the propensity score models were binary indicators coded as present or absent and did not contain missing data. Missingness ranged from <1% for maternal age to approximately 13% for BMI, primarily because BMI was unavailable in the dataset for study years 2005 and 2006. This missingness in BMI was not related to participant characteristics or exposure/outcome status. For those 2 years, BMI information therefore did not contribute to propensity score estimation.

For cells with fewer than 11 events, numbers were not reported to comply with the confidentiality requirements of HCAI; and aRRs were not calculated. All analyses were performed using Statistical Analysis Software V.9.4.

### Patients and public

Patients or the public were not involved in the design, or conduct, or reporting, or dissemination plans of our research.

## Results

The sample included 8188 liveborn singletons from women with SLE delivered between 2005 and 2020. A majority of the women were Hispanic (43.9%), followed by non-Hispanic white (28.5%). Hispanic and non-Hispanic black women more often had public insurance (51.4% and 52.2%) compared with Asian and non-Hispanic white women (15.4% and 23.9%), respectively, and a higher proportion of women with education at or below 12th grade. Close to 30% of non-Hispanic black women had a prepregnancy BMI of ≥30, and they also had the highest prevalence of hypertensive disorders. In this cohort, Asian women had the highest proportion of SLE with LN (17.4%), compared with 4.5% of white women and approximately 12.5% in Hispanic and black women (table 1).

**Table 1:** Baseline sociodemographic characteristics and clinical comorbidities compared between racial and ethnic groups in pregnant individuals with SLE (n=8188), 2005–2020

	Asian	Hispanic	Non-Hispanic black	Others§	Non-Hispanic white	P value
	n (%)	n (%)	n (%)	n (%)	n (%)	
Sample	1095	3600	782	377	2334	
Maternal age (years)						**<0.0001**
<18	*	†	*	*	*	
18–34	696 (63.6)	2842 (78.9)	592 (75.7)	288 (76.4)	1600 (68.6)	
35–44	395 (36.1)	724 (20.1)	180 (23.0)	87 (23.1)	715 (30.6)	
≥45	*	*	*	*	*	
Expected payer						**<0.0001**
Private	862 (78.7)	1571 (43.6)	321 (41.1)	194 (51.4)	1651 (70.7)	
Public	169 (15.4)	1849 (51.4)	408 (52.2)	166 (44.0)	557 (23.9)	
Self-pay	18 (1.6)	26 (0.7)	*	*	*	
Other	46 (4.2)	154 (4.3)	†	†	†	
Maternal education						**<0.0001**
≤12	126 (11.5)	1774 (49.3)	285 (36.5)	†	520 (22.3)	
>12	942 (86.0)	1754 (48.7)	483 (61.8)	260 (69.0)	1778 (76.2)	
Missing	27 (2.5)	72 (2.0)	14 (1.8)	*	36 (1.5)	
Nulliparity	566 (51.7)	1228 (34.1)	259 (33.1)	142 (37.7)	952 (40.8)	**<0.0001**
Prepregnancy BMI						**<0.0001**
<18.5	94 (8.6)	99 (2.8)	31 (4.0)	18 (4.8)	97 (4.2)	
18.5 to <25	640 (58.5)	1210 (33.6)	242 (31.0)	146 (38.7)	1049 (44.9)	
25 to <30	167 (15.3)	931 (25.9)	170 (21.7)	90 (23.9)	427 (18.3)	
≥30	80 (7.3)	913 (25.4)	234 (29.9)	89 (23.6)	409 (17.5)	
Missing‡	114 (10.4)	447 (12.4)	105 (13.4)	34 (9.0)	352 (15.1)	
Hypertension						**<0.0001**
None	815 (74.4)	2625 (72.9)	498 (63.7)	272 (72.2)	1880 (80.6)	
Pre-existing	94 (8.6)	298 (8.3)	115 (14.7)	33 (8.8)	146 (6.3)	
Gestational	38 (3.5)	150 (4.2)	44 (5.6)	16 (4.2)	117 (5.0)	
Pre-eclampsia	148 (13.5)	527 (14.6)	125 (16.0)	56 (14.9)	191 (8.2)	
Diabetes mellitus						**0.0003**
None	960 (87.7)	3166 (87.9)	685 (87.6)	341 (90.5)	2105 (90.2)	
Pre-existing	16 (1.5)	84 (2.3)	33 (4.2)	*	45 (1.9)	
Gestational	119 (10.9)	350 (9.7)	64 (8.2)	†	184 (7.9)	
Hyperlipidaemia	13 (1.2)	53 (1.5)	11 (1.4)	*	18 (0.8)	0.1378
Smoking during pregnancy	11 (1.0)	113 (3.1)	62 (7.9)	41 (10.9)	181 (7.8)	**<0.0001**
Alcohol use during pregnancy	*	19 (0.5)	*	*	23 (1.0)	**0.0040**
Drug use during pregnancy	*	124 (3.4)	67 (8.6)	29 (7.7)	123 (5.3)	**<0.0001**
Depression	40 (3.7)	215 (6.0)	71 (9.1)	33 (8.8)	215 (9.2)	**<0.0001**
SLE disease characteristics						
SLE with LN	190 (17.4)	441 (12.3)	98 (12.5)	35 (9.3)	105 (4.5)	**<0.0001**
SLE with APS	65 (5.9)	228 (6.3)	50 (6.4)	22 (5.8)	121 (5.2)	0.0965

Bold when p<0.05.

* Not displayed when n<11.

† Value masked to prohibit cell with n<11 calculation.

‡ BMI is not available for 2005 and 2006 births.

§ Other race includes American Indian/Alaska Native, Native Hawaiian/Pacific Islander, other race, two or more races.

APS, antiphospholipid syndrome; BMI, body mas index; LN, lupus nephritis.

### Non-Hispanic black individuals with SLE experienced the highest burden of maternal CVEs and APOs

Non-Hispanic black individuals had the highest occurrence of CVEs during pregnancy (4.0%) compared with 1.6–2.0% among other groups. Compared with non-Hispanic white individuals, the aRR of CVEs was 1.6 (95% CI 0.9 to 2.6) for black women and below 1.0 for other racial/ethnic groups (Asian: aRR 0.8, 95% CI 0.4 to 1.4; Hispanic: aRR 0.8, 95% CI 0.5 to 1.2), although the difference was not statistically significant (table 2). Non-Hispanic black individuals also had the highest occurrence of composite maternal APOs (24.6%) and infant APOs (54.6%) compared with 13.5–17.2% maternal and 34.6–49.4% infant APOs in other racial/ethnic groups. Compared with non-Hispanic white women, only non-Hispanic black women had a significantly elevated risk of any maternal APO (aRR: 1.3, 95% CI 1.1 to 1.6), whereas the adjusted risk of any infant APO was higher among Asian (aRR 1.3, 95% CI 1.2 to 1.5), Hispanic (aRR 1.2, 95% CI 1.1 to 1.3) and non-Hispanic black women (aRR 1.4, 95% CI 1.2 to 1.6) (table 2).

**Table 2:** Cardiovascular events (CVEs) and adverse pregnancy outcomes (APOs) among racial/ethnic groups compared to non-Hispanic white individuals with SLE (n=8188), 2005–2020

	Asian	Hispanic	Non-Hispanic black	Others	Non-Hispanic white
Sample	1095	3600	782	377	2334
CVE*					
No CVE (n %)	1077 (98.4)	3528 (98.0)	751 (96.0)	370 (98.1)	2291 (98.2)
					Reference
Any CVE (n %)	18 (1.6)	72 (2.0)	31 (4.0)	†	43 (1.8)
RR (95% CI)	0.9 (0.5 to 1.5)	1.1 (0.7 to 1.6)	2.2 (1.4 to 3.4)	†	
aRR (95% CI)	0.8 (0.4 to 1.4)	0.8 (0.5 to 1.2)	1.6 (0.9 to 2.6)	†	
VTE CVE (DVT or PE) (n %)	†	32 (0.9)	13 (1.7)	†	19 (0.8)
RR (95% CI)	†	1.1 (0.6 to 1.9)	2.1 (1.0 to 4.2)	†	
aRR (95% CI)	†	0.8 (0.4 to 1.5)	1.7 (0.8 to 3.6)	†	
Non-VTE CVE (n %)	‡	41 (1.1)	20 (2.6)	†	26 (1.1)
RR (95% CI)	1.1 (0.6 to 2.2)	1.0 (0.6 to 1.7)	2.3 (1.3 to 4.1)	†	
aRR (95% CI)	0.9 (0.5 to 1.9)	0.7 (0.4 to 1.2)	1.5 (0.8 to 2.9)	†	
Maternal APOs					
No maternal APO (n %)	947 (86.5)	3002 (83.4)	590 (75.5)	312 (82.8)	2017 (86.4)
					Reference
Any maternal APO (n %)	148 (13.5)	598 (16.6)	192 (24.6)	65 (17.2)	317 (13.6)
RR (95% CI)	1.0 (0.9 to 1.2)	1.2 (1.1 to 1.4)	1.8 (1.5 to 2.2)	1.3 (1.0 to 1.7)	
aRR (95% CI)	1.0 (0.8 to 1.2)	1.0 (0.9 to 1.2)	1.3 (1.1 to 1.6)	1.0 (0.8 to 1.3)	
Non-cardiac severe maternal morbidity (n %)	39 (3.6)	119 (3.3)	43 (5.5)	11 (2.9)	58 (2.5)
RR (95% CI)	1.3 (1.0 to 2.2)	1.3 (1.0 to 1.8)	2.2 (1.5 to 3.3)	1.2 (0.6 to 2.2)	
aRR (95% CI)	1.0 (0.6 to 1.5)	0.9 (0.6 to 1.2)	1.3 (0.8 to 2.0)	0.7 (0.4 to 1.4)	
Maternal intensive care use at delivery (n %)	†	18 (0.5)	†	†	†
RR (95% CI)	†	2.9 (1.0 to 8.6)	†	†	
aRR (95% CI)	†	1.6 (0.5 to 5.3)	†	†	
Maternal readmission in first year (n %)	124 (11.3)	537 (14.9)	171 (21.9)	57 (15.1)	290 (12.4)
RR (95% CI)	0.9 (0.7 to 1.1)	1.2 (1.0 to 1.4)	1.8 (1.5 to 2.1)	1.2 (0.9 to 1.6)	
aRR (95% CI)	1.0 (0.8 to 1.2)	1.0 (0.9 to 1.2)	1.3 (1.0 to 1.6)	1.0 (0.7 to 1.3)	
Maternal death in first year (n %)	†	†	†	†	†
RR (95% CI)	†	†	†	†	
aRR (95% CI)	†	†	†	†	
Infant APOs					
No infant APO (n %)	554 (50.6)	1955 (54.3)	363 (46.4)	223 (59.2)	1527 (65.4)
					Reference
Any infant APO (n %)	541 (49.4)	1645 (45.7)	419 (54.6)	154 (40.9)	807 (34.6)
RR (95% CI)	1.4 (1.3 to 1.6)	1.3 (1.2 to 1.4)	1.5 (1.4 to 1.7)	1.2 (1.0 to 1.4)	
aRR (95% CI)	1.3 (1.2 to 1.5)	1.2 (1.1 to 1.3)	1.4 (1.2 to 1.6)	1.1 (0.9 to 1.3)	
Preterm birth (gestation<37 weeks) (n %)	251 (22.9)	844 (23.4)	230 (29.4)	68 (18.0)	355 (15.2)
RR (95% CI)	1.5 (1.3 to 1.8)	1.5 (1.4 to 1.7)	1.9 (1.6 to 2.3)	1.2 (0.9 to 1.5)	
aRR (95% CI)	1.2 (1.1 to 1.5)	1.2 (1.1 to 1.4)	1.5 (1.3 to 1.8)	1.0 (0.8 to 1.3)	
Early (<32 WGA) (n %)	57 (5.2)	191 (5.3)	64 (8.2)	‡	49 (2.1)
RR (95% CI)	2.6 (1.8 to 3.8)	2.7 (2.0 to 3.7)	4.3 (3.0 to 6.2)	1.8 (1.0 to 3.2)	
aRR (95% CI)	1.7 (1.1 to 2.5)	1.5 (1.1 to 2.1)	2.3 (1.5 to 3.4)	1.1 (0.6 to 2.0)	
Moderate (32 to <34 WGA) (n %)	29 (2.7)	122 (3.4)	22 (2.8)	†	31 (1.3)
RR (95% CI)	2.2 (1.3 to 3.6)	2.7 (1.9 to 4.1)	2.5 (1.4 to 4.3)	†	
aRR (95% CI)	1.4 (0.8 to 2.5)	1.8 (1.2 to 2.8)	1.7 (0.9 to 3.0)	†	
Late (34 to <37 WGA) (n %)	165 (15.1)	531 (14.8)	144 (18.4)	47 (12.5)	275 (11.8)
RR (95% CI)	1.3 (1.1 to 1.6)	1.3 (1.1 to 1.5)	1.7 (1.4 to 2.1)	1.1 (0.8 to 1.5)	
aRR (95% CI)	1.2 (1.0 to 1.5)	1.1 (1.0 to 1.3)	1.4 (1.1 to 1.8)	0.9 (0.7 to 1.3)	
Small-for-gestational age infant (n %)	272 (24.8)	647 (18.0)	166 (21.2)	53 (14.1)	289 (12.4)
RR (95% CI)	2.0 (1.7 to 2.4)	1.5 (1.3 to 1.7)	1.7 (1.4 to 2.1)	1.1 (0.8 to 1.5)	
aRR (95% CI)	1.7 (1.5 to 2.1)	1.4 (1.2 to 1.6)	1.6 (1.3 to 1.9)	1.0 (0.8 to 1.4)	
Neonatal intensive care use at birth (n %)	159 (14.5)	455 (15.7)	153 (19.6)	59 (15.7)	246 (10.5)
RR (95% CI)	1.4 (1.1 to 1.7)	1.5 (1.3 to 1.7)	1.9 (1.5 to 2.3)	1.5 (1.1 to 2.0)	
aRR (95% CI)	1.2 (0.9 to 1.4)	1.2 (1.0 to 1.4)§	1.4 (1.1 to 1.7)	1.2 (0.9 to 1.6)	
Infant readmission in the first year (n %)	114 (10.4)	477 (13.3)	101 (12.9)	47 (12.5)	241 (10.3)
RR (95% CI)	1.0 (0.8 to 1.3)	1.3 (1.1 to 1.5)	1.3 (1.0 to 1.6)	1.2 (0.9 to 1.7)	
aRR (95% CI)	1.0 (0.8 to 1.3)	1.2 (1.0 to 1.4)§	1.1 (0.8 to 1.4)	1.1 (0.8 to 1.5)	
Infant death in the first year (n %)	†	31 (0.9)	14 (1.8)	†	13 (0.6)
RR (95% CI)	†	1.5 (0.8 to 3.0)	3.2 (1.5 to 6.8)	†	
aRR (95% CI)	†	1.1 (0.5 to 2.1)	2.2 (1.0 to 5.1)	†	

Adjusted relative risk (aRR) adjusted for racial/ethnicity propensity score created from age group, primary payer, maternal education, prepregnancy body mass index group, clinical comorbidities (pre-existing and gestational diabetes mellitus and hypertension, pregnancy-induced hypertension, hyperlipidaemia, depression, alcohol, tobacco and drug use during pregnancy), presence of concurrent lupus nephritis and presence of antiphospholipid syndrome.

Comparison group (reference): non-Hispanic white individuals.

* CVE was defined as a composite of acute myocardial infarction, stroke, heart failure and peripartum cardiomyopathy, myocarditis, pericarditis, dysrhythmia, and (VTE) [PE or DVT].

† not displayed and risk not calculated when n < 11.

‡ Value masked to prohibit cell with n<11 calculation.

§ p <0.05

DVT, deep vein thrombosis; PE, pulmonary embolism ; SMM, severe maternal morbidity; VTE, venous thromboembolism ; WGA, weeks of gestational age.

Infants born to non-Hispanic black women had a higher infant death rate (1.8%) compared with those born to non-Hispanic white (0.6%) and Hispanic (0.9%) individuals. PTB was highest among black individuals (29.4%) compared with 15.2% of white individuals, whereas Asian individuals had the highest prevalence of SGA infants (24.8%) compared with 12.4% among white individuals (table 2). Among those for whom the indication for PTB could be reasonably ascertained, both spontaneous and provider-initiated PTB were more frequent among non-Hispanic black individuals (20.2% vs 10.1% for spontaneous PTB and 7.2% vs 4.5% for provider-initiated PTB, respectively). Compared with non-Hispanic white women, Asian (aRR 1.2, 95% CI 1.1 to 1.5), Hispanic (aRR 1.2, 95% CI 1.1 to 1.4) and non-Hispanic black (aRR 1.5, 95% CI 1.3 to 1.8) women had a higher risk of PTB, with the risk of early PTB (<32 WGA) being highest among non-Hispanic black individuals (aRR 2.3, 95% CI 1.5 to 3.4). SGA births were higher for Asian (aRR 1.7, 95% CI 1.5 to 2.1), Hispanic (1.4, 95% CI 1.2 to 1.6) and non-Hispanic black (aRR 1.6, 95% CI 1.3 to 1.9) women compared with non-Hispanic white women with SLE (table 2).

In stratified analysis by CVE status, the composite of maternal and infant APOs remained higher in non-Hispanic black women, with or without a CVE. Without CVE, the point estimates for maternal and infant APOs were higher by ~10 and 18 percentage points compared with non-Hispanic white women (23.4% vs 13.4% and 52.3% vs 34.1%, respectively). With CVEs, the point estimates for maternal and infant APOs were even higher (51.6% vs 25.6% for maternal APOs and 83.9% vs 58.1% for infant APOs). There was some evidence for effect modification by CVE status for maternal APOs. For example, in the CVE stratum, aRRs of 1.8 (95% CI 0.8 to 4.1) and 1.7 (95% CI 0.7 to 4.1) were calculated for Hispanic and black individuals, respectively, compared with the non-Hispanic white group (table 3), versus aRRs in the corresponding racial/ethnic groups of 1.0 and 1.3 in the unstratified analysis. No such differences were evident in the no CVE stratum or for infant APOs. However, small sample sizes in the CVE stratum limited precise adjusted risk estimation and yielded wide CIs for adjusted risk estimates (table 3).

**Table 3:** Adverse pregnancy outcomes (APOs) among racial/ethnic groups compared to non-Hispanic white individuals with SLE, stratified by CVE status, 2005–2020

	Asian	Hispanic	Non-Hispanic black	Others	Non-Hispanic white
					Reference
SLE with CVE during pregnancy					
Sample	18	72	31	*	43
Any maternal APO (n %)	*	31 (32.1)	16 (51.6)	*	11 (25.6)
RR (95% CI)	*	1.7 (0.8 to 3.3)	2.0 (0.9 to 4.3)	*	
aRR (95% CI)	*	1.8 (0.8 to 4.1)	1.7 (0.7 to 4.1)	*	
Any infant APO (n %)	14 (77.8)	51 (70.8)	26 (83.9)	*	25 (58.1)
RR (95% CI)	1.3 (0.7 to 2.6)	1.2 (0.8 to 2.0)	1.4 (0.8 to 2.5)	*	
aRR (95% CI)	1.2 (0.5 to 2.9)	1.2 (0.7 to 2.1)	1.3 (0.7 to 2.4)	*	
SLE without CVE during pregnancy					
Sample	1077	3528	751	370	2291
Any maternal APO (n %)	139 (12.9)	567 (16.1)	176 (23.4)	63 (17.0)	306 (13.4)
RR (95% CI)	1.0 (0.8 to 1.2)	1.2 (1.0 to 1.4)	1.8 (1.5 to 2.1)	1.1 (0.9 to 1.4)	
aRR (95% CI)	1.0 (0.8 to 1.2)	1.0 (0.9 to 1.2)	1.3 (1.1 to 1.6)	1.0 (0.7 to 1.3)	
Any infant APO (n %)	527 (48.9)	1594 (45.2)	393 (52.3)	150 (40.5)	782 (34.1)
RR (95% CI)	1.4 (1.3 to 1.6)	1.3 (1.2 to 1.4)	1.5 (1.4 to 1.7)	1.2 (1.0 to 1.4)	
aRR (95% CI)	1.3 (1.2 to 1.5)	1.2 (1.1 to 1.3)	1.4 (1.2 to 1.6)	1.1 (0.9 to 1.3)	

Comparison group (reference): non-Hispanic white individuals.

aRR adjusted for racial/ethnicity propensity score created from age group, primary payer, maternal education, prepregnancy body mass index group, clinical comorbidities (pre-existing and gestational diabetes mellitus and hypertension (HTN), pregnancy-induced HTN, hyperlipidaemia, depression, alcohol, tobacco and drug use during pregnancy), presence of LN, presence of antiphospholipid syndrome.

* Not displayed and risk not calculated when n<11.

aRR, adjusted relative risk; CVE, cardiovascular event.

### CVEs were associated with higher risks of select maternal and infant APOs across racial groups

Within racial/ethnic groups, the presence of CVEs was associated with elevated risks for composite maternal APOs among Hispanic (aRR 1.7, 95% CI 1.1 to 2.5), non-Hispanic black (aRR 1.8, 95% CI 1.0 to 3.0) and non-Hispanic white (aRR 1.8, 95% CI 1.0 to 3.2) individuals. The risk of non-cardiac SMM was 2.7-fold (95% CI 1.3 to 5.6) for Hispanic women with a CVE during pregnancy compared with those without a CVE. The presence of CVE was not significantly associated with any infant APOs in any racial/ethnic group, except for a modestly increased risk in the non-Hispanic white group where the aRR was 1.6 (95% CI 1.1 to 2.4). The presence of a CVE was associated with a 1.9-fold (95% CI 1.0 to 3.5) increase in PTB risk among Asian women and a 2.8-fold (95% CI 1.6 to 4.8) increase in the risk of neonatal intensive care unit (NICU) use for non-Hispanic white women. Risk estimates for most outcomes and subgroups were limited by sample sizes (table 4).

**Table 4:** Risk of adverse pregnancy outcomes (APOs) among individuals with SLE with a CVE versus without a CVE, stratified by racial/ethnicity, 2005–2020

	Asian (n=1095)	Hispanic (n=3600)	Non-Hispanic black (n=782)	Non-Hispanic white (n=2334)
Any maternal APO				
RR (95% CI)	*	2.7 (1.9 to 3.8)	2.2 (1.3 to 3.7)	1.9 (1.0 to 3.5)†
aRR (95% CI)	*	1.7 (1.1 to 2.5)	1.8 (1.0 to 3.0)†	1.8 (1.0 to 3.2)
Non-cardiac severe maternal morbidity (SMM‡)				
RR (95% CI)	*	5.0 (2.7 to 9.3)	*	*
aRR (95% CI)	*	2.7 (1.3 to 5.6)	*	*
Acute renal failure				
RR (95% CI)	*	*	*	*
aRR (95% CI)	*	*	*	*
Blood transfusion				
RR (95% CI)	*	*	*	*
aRR (95% CI)	*	*	*	*
Maternal readmission in first year				
RR (95% CI)	*	2.2 (1.4 to 3.3)	1.7 (0.9 to 3.1)	*
aRR (95% CI)	*	1.3 (0.8 to 2.1)	1.4 (0.7 to 2.6)	*
Any infant APO				
RR (95% CI)	1.6 (0.9 to 2.7)	1.6 (1.2 to 2.1)	1.6 (1.1 to 2.4)	1.7 (1.1 to 2.5)
aRR (95% CI)	1.2 (0.7 to 2.1)	1.2 (0.9 to 1.6)	1.4 (0.9 to 2.1)	1.6 (1.1 to 2.4)
Preterm birth/gestation<37 weeks				
RR (95% CI)	3.3 (1.9 to 5.7)	2.0 (1.4 to 2.8)	2.2 (1.4 to 3.5)	*
aRR (95% CI)	1.9 (1.0 to 3.5)†	1.1 (0.8 to 1.7)	1.7 (1.1 to 2.8)	*
Small-for-gestational age infant				
RR (95% CI)	*	1.6 (1.0 to 2.4)†	*	*
aRR (95% CI)	*	1.2 (0.8 to 2.0)	*	*
Neonatal intensive care use at birth				
RR (95% CI)	*	2.2 (1.4 to 3.3)	2.2 (1.3 to 4.0)	3.0 (1.7 to 5.2)
aRR (95% CI)	*	1.2 (0.7 to 1.8)	1.6 (0.9 to 3.0)	2.8 (1.6 to 4.8)
Infant readmission in first year				
RR (95% CI)	*	*	*	*
aRR (95% CI)	*	*	*	*

Other race includes American Indian/Alaska Native, Native Hawaiian/Pacific Islander, other race, two or more races.

Comparison group (reference): SLE without CVEs within each racial/ethnic group.

aRR adjusted for propensity score for CVE created from age group, primary payer, maternal education, prepregnancy BMI group, clinical comorbidities (pre-existing and gestational diabetes mellitus and hypertension (HTN), pregnancy-induced HTN, hyperlipidaemia, depression, alcohol, tobacco and drug use during pregnancy), presence of LN and presence of antiphospholipid syndrome.

* Not calculated when n<11.

† p<0.05.

‡ Non-cardiac components of SMM include acute renal failure, acute respiratory distress syndrome, disseminated intravascular coagulation, amniotic fluid embolism, blood transfusion, severe anesthesia complications, sepsis, hysterectomy, temporary tracheostomy and ventilation.

aRR, adjusted relative risk; CVE, cardiovascular event; SMM, severe maternal morbidity.

### Maternal CVEs and APOs among nulliparous women

On restricting the analyses to nulliparous women, no significant differences in CVE risk were observed for any of the four racial/ethnic groups when compared with non-Hispanic white women. Composite maternal and infant APOs were more frequent among non-Hispanic black women (27.5% and 57.9%), corresponding to an aRR of 1.9 (95% CI 1.3 to 2.6) for maternal APOs and 1.6 (95% CI 1.3 to 1.9) for infant APOs relative to non-Hispanic white women. Non-Hispanic black women had a higher occurrence of PTB than non-Hispanic white women (30.9% vs 12.6%), whereas Asian individuals had the highest occurrence of SGA infants (27.6% vs 14.3%) (online supplemental table 3). CVE-stratified analyses for nulliparous women were limited by small sample size. Among pregnancies without CVEs, non-Hispanic black women had the highest occurrence of composite maternal and infant APOs (26.6% and 57.1%, respectively), with adjusted risks of 1.8 (95% CI 1.3 to 2.5) and 1.6 (95% CI 1.3 to 1.9) compared with non-Hispanic white women; these estimates were slightly higher than those observed in the overall analysis not restricted by parity (aRRs of 1.3 and 1.4, respectively) (online supplemental tables 3 and 4). Among nulliparous Asian women, the presence of CVEs was associated with 29.3-fold (95% CI 7.1 to 121.0) risk of acute renal failure and 7.8-fold (2.3 to 26.7) risk of non-cardiac SMM, although these estimates were imprecise because of small sample size and wide CI. Overall, CVEs were not strongly associated with infant APOs across racial/ethnic groups (online supplemental table 5).

## Discussion

Our study using a large population-based California birth cohort of 8188 liveborn singletons to women with SLE delivered between 2005 to 2020, highlights three principal findings. Non-Hispanic black individuals with SLE bore the highest absolute burden of both maternal CVEs and APOs. The presence of a CVE during pregnancy was associated with a higher occurrence of maternal APOs across racial/ethnic groups compared with non-Hispanic white women, whereas associations with infant APOs were smaller and more variable, suggesting that a CVE may represent a broadly relevant risk factor for APOs among women with SLE. Analyses restricted to nulliparous individuals yielded broadly similar results. Importantly, attenuation of many of the crude associations after adjustment suggested that differences in sociodemographic and clinical characteristics explained part of the crude between-group differences. In our cohort, non-Hispanic black individuals more often had lower socioeconomic status, higher prevalence prepregnancy BMI of ≥30, and the highest prevalence of hypertensive disorders.

The higher burden of CVEs and APOs among non-Hispanic black women aligns with large US studies demonstrating significantly higher risks of chronic hypertension, pre-eclampsia, PTB and fetal growth restriction among black individuals with or without SLE, compared with white women.^8 9 14^ Our findings are also in line with prior work in the same birth cohort, in which non-Hispanic black women with rheumatoid arthritis had a 2.0 to 2.4 times higher risks of PTB than Asian, Hispanic or non-Hispanic white women.^15^ While race/ethnicity-stratified data on maternal CVEs in SLE are not available, black women with SLE have a higher lifetime risk of cardiovascular diseases, including ischaemic heart diseases, cerebrovascular diseases and peripheral vascular diseases.^16^ Taken together, these observations indicate that racial/ethnic differences we observed in SLE pregnancies parallel the disparities in the general obstetric population but occur in the context of substantially higher absolute risks of maternal and infant APOs and CVEs conferred by SLE.^1–4^

Differences in CVEs and APOs likely reflect not only the differences in measured comorbidity burden and SLE disease-related factors but also the effect of other unmeasured structural and social influences such as healthcare access, insurance coverage, care fragmentation and the cumulative stress of discrimination. While our study was not able to assess for these factors, research from a US military health system which modelled a universal healthcare access cohort found no significant racial differences in SMM for deliveries complicated by pre-eclampsia, however, black women still had higher absolute risks of SMM compared with white women.^17^ This suggests that improving care delivery factors may reduce disparities in some outcomes but not eliminate the differences. Our study also does not capture self-reported experiences of racial discrimination, which have been associated with greater SLE activity and organ damage.^18^ Additionally, the nature of the database limited our ability to capture several important confounders, including disease duration, disease activity or medication exposures before or during pregnancy and healthcare access including the adequacy of prenatal care. While racial/ethnic-stratified data on the prenatal care and medication adherence during pregnancy are limited, racial/ethnic differences in SLE disease severity and accrual of organ damage^5–7^ and medication adherence^19^ have been well described and likely extend into preconception and pregnancy periods. Additionally, studies in the general obstetric population suggest that non-Hispanic black women have increased likelihood of receiving inadequate prenatal care and experiencing APOs.^14^ Together, these findings underscore the need for future work to evaluate whether access to high-quality preconception care and guideline-concordant management of SLE during pregnancy differ by race/ethnicity and contribute to the observed disparities.

Unlike other outcomes, which were more frequent among black individuals, Asian women had the highest prevalence of SGA infants. CVEs were associated with higher occurrence of maternal APOs across all groups. Among nulliparous pregnancies, Asian women showed the most pronounced RRs of certain APOs when associated with CVEs (eg, 29.3-fold risk of acute renal failure (95% CI 7.1 to 121.0) and 7.8-fold risk of non-cardiac SMM (95% CI 2.3 to 26.7)), although these estimates were imprecise because of limited sample size and wide CIs.

Prior studies provide relevant clinical context on the effect modification by race/ethnicity. One study using the US National Inpatient Sample database found that black women had the highest prevalence of pre-eclampsia and highest absolute numbers of maternal CVEs and other APOs when associated with pre-eclampsia; however, Asian/Pacific Islander women with versus without pre-eclampsia had the largest relative increase in the risk of cardiovascular complications.^20^ Another study showed that chronic hypertension amplified stroke risk during delivery in all groups with the largest relative increase among Asian/Pacific Islander women (1.71, 1.75 and 3.62-fold higher stroke risk for black, Hispanic and Asian/Pacific Islander women with chronic hypertension, respectively).^21^

In this study, CVEs during pregnancy were associated with higher occurrence of maternal APOs across racial/ethnic groups. Of note, in the stratified analysis within race/ethnic group, infants born to non-Hispanic white women with a CVE in pregnancy had the highest adjusted RR of any infant APO (aRR 1.6, 95% CI 1.1 to 2.4) and specifically admission to the NICU (aRR 2.8, 95% CI 1.6 to 4.8), consistent with the finding that CVEs introduced elevated risks irrespective of race/ethnic group. CVEs during pregnancy likely reflect broader underlying systemic cardiovascular dysfunction. Maternal cardiovascular diseases have also been linked to increased risk of APOs including maternal mortality, maternal cardiac, pulmonary and cerebrovascular events, PTB, SGA infants and perinatal deaths.^22 23^ Ensuring access to prenatal care for all women with SLE, coupled with systematic cardiovascular risk assessment and optimisation of modifiable cardiovascular risk factors may reduce both APO and CVE risks. For high-risk patients, co-management by a multidisciplinary team consisting of rheumatology, maternal-fetal medicine and cardiology may further improve outcomes.

The strengths of our study include the use of a large, population-based statewide birth cohort created by linking vital statistics to maternal and infant hospital encounter data over a 15-year period. This approach enabled us to comprehensively study CVEs and a broad range of maternal and infant APOs across racial and ethnic groups, uncovering clinically meaningful differences in outcomes. The use of a statewide cohort also improves the generalisability of our findings. An important limitation was potential misclassification of SLE related to reliance on a single ICD code from an administrative database, as has been shown in prior validation studies. However, the SOMI database used for this study is structurally different from most longitudinal claims datasets in that it captures only inpatient, emergency department and ambulatory surgery encounters from 1 year before to 1 year after delivery, primarily captured during delivery admission, and validation studies for this scenario are lacking. In this context, requiring>1 SLE codes could improve specificity but could exclude true SLE cases who did not have repeated hospital-based encounters in the year surrounding delivery, preferentially retaining those with more severe disease. Furthermore, misclassification of SLE status is unlikely to vary systematically by race/ethnicity and is unlikely to fully account for the observed racial/ethnic differences.

Other limitations include underascertainment of certain covariates including cardiac risk factors, including alcohol, tobacco and drug use, when relying on ICD codes from discharge data. We also lacked granular information on disease activity, chronicity, autoantibody profiles, prior adverse outcomes, access to preconception care and medication use or adherence. Although the propensity score approach allowed adjustment for a broad range of sociodemographic, clinical and SLE-related variables, residual confounding by unmeasured social and clinical factors, such as medication adherence, remains possible. These factors may differ across racial/ethnic groups and partly contribute to the observed associations. For example, persistently low hydroxychloroquine concentrations during pregnancy have been associated with higher odds of PTB, and prior studies have reported higher risk of nonadherence among black and Hispanic individuals compared with white individuals.^24 25^ Incomplete adjustment may therefore have contributed to overestimation of some racial/ethnic differences. Missing covariate data, particularly for BMI during years 2005–2006, may also have affected propensity score estimation. However, individuals with missing covariate data were retained in the analyses, and propensity scores incorporated the remaining available covariate information for those individuals.

Records in the SOMI database are structured by infant rather than mother; thus, each pregnancy is captured as an independent record without linkage across pregnancies, limiting ascertainment of prior history of CVEs or APOs; therefore, residual confounding by these variables is possible. SOMI contains information on liveborn singletons but does not capture important obstetric variables such as prior history of recurrent pregnancy losses, which may potentially underestimate the full burden of APOs and CVEs across SLE pregnancies. Importantly, due to the nature of the database used, diagnoses were primarily ascertained from acute care encounters such as emergency department and hospitalisation records, which limited our ability to determine the temporal sequence of CVEs and APOs during pregnancy. Certain APOs, such as SGA likely reflect pathophysiologic processes that begin well before their diagnosis at delivery; therefore, observed associations with maternal CVEs are more likely to reflect the contribution from shared underlying risk factors than a causal relationship. For other APOs such as pre-eclampsia, a reverse causation is more plausible, as maternal CVEs may precede and potentially contribute to the diagnosis of pre-eclampsia. In addition, reliance on data from acute care settings records may have led to under-ascertainment of CVEs and APOs that were managed in the outpatient setting or not coded during hospitalisation, which may have affected the observed associations. However, many of the CVEs and APOs examined in this study would be expected to come to clinical attention in acute care settings or at the time of delivery admission. The direction of potential bias related to outcome underascertainment is uncertain, particularly if ascertainment differed across racial/ethnic groups or patterns of healthcare utilisation. Because of the observational nature of the data and the limitations described, these findings should be interpreted as descriptive and hypothesis-generating. Future studies with prospective ascertainment of outcomes, for example, serial ultrasound assessments to detect intrauterine growth restriction and systematic recording of pre-eclampsia features and maternal SMM indicators (eg, sepsis, renal failure) in relation to timing of CVEs will be important to clarify these relationships.

In conclusion, this study revealed clinically relevant racial and ethnic differences in CVEs and APOs among individuals with SLE. Non-Hispanic black women experienced the highest absolute burden of maternal CVEs, maternal APOs and infant APOs, mirroring the observed patterns in the general obstetric population, but occurring in the setting of an already elevated baseline risks associated with SLE. Across racial/ethnic groups, the presence of maternal CVEs was associated with higher occurrence of maternal APOs. The attenuation of many observed associations after adjustment for covariates indicates that interpreting race/ethnicity effects in SLE pregnancies requires acknowledging the layered contributions of sociodemographic and clinical factors. These findings underscore the importance of proactively screening for pregnancy complications and CVEs in all SLE pregnancies, while also addressing the broader social drivers of health that contribute to such differences. Multidisciplinary cardio-obstetric care models involving rheumatology, maternal-fetal medicine and cardiology may help improve risk stratification and pregnancy management, providing a framework to translate these observational findings into improved and more equitable maternal and child health outcomes.

## Supplementary material

10.1136/lupus-2025-001846 online supplemental table 1

10.1136/lupus-2025-001846 online supplemental table 2

10.1136/lupus-2025-001846 online supplemental table 3

10.1136/lupus-2025-001846 online supplemental table 4

10.1136/lupus-2025-001846 online supplemental table 5

## Data Availability

All data relevant to the study are included in the article or uploaded as supplementary information.
