# Supplementary material for: Racial differences in cardiovascular events and adverse pregnancy outcomes among pregnant individuals with SLE
Source: Lupus Sci Med. 2026 Sep 15;13(2):e001846. doi: 10.1136/lupus-2025-001846 (PMC13583854; doi:10.1136/lupus-2025-001846)
Supplement: Supplementary file 1 [file lupus-13-2-s001.docx]

| **Supplementary Table 1**. ICD codes used to identify cardiovascular events (CVEs) and cardiovascular risk factors. | | |
| --- | --- | --- |
|  | **ICD-9 codes** | **ICD-10 codes** |
| **Cardiovascular events (CVEs)** | | |
| **Acute myocardial infarction (AMI)** | 410 | I21, I22 |
| **Acute cerebrovascular accidents** | | |
| Hemorrhagic stroke | 430, 431 | I60, I61 |
| Ischemic stroke | 433.x1 (433.01, 433.11, 433.21, 33.31, 433.81, 433.91), 434.x1 (434.01, 434.11, 434.91) | I63 |
| **Heart failure (acute or unspecified, not rheumatic)** | 402.01, 402.11, 402.91, 404.01, 404.03, 404.11, 404.13, 404.91, 404.93, 428.0, 428.1, 428.20, 428.21, 428.23, 428.30, 428.31, 428.33, 428.40, 428.41, 428.43, 428.9 | I11.0, I13.0, I13.2, I50.1, I50.20, I50.21, I50.23  I50.30, I50.31, I50.33, I50.40, I50.41, I50.43  I50.8x (except I50.812), I50.9 |
| **Peripartum cardiomyopathy (PPCM)** | 674.5x (674.50 - 674.54) | O90.3 |
| **Inflammatory heart diseases** | | |
| Acute pericarditis | 420 | I30.0, I30.8, I30.9, I32 |
| Acute myocarditis (not septic or toxic) | 422.0, 422.90, 422.91, 422.99, 429.0 | I40.1, I40.8, I40.9, I41, I51.4 |
| **Cardiac dysrhythmias** | | |
| Atrial fibrillation/flutter | 427.3x (427.31, 427.32) | I48 (I48.0, I48.11, I48.19, I48.20, I48.21, I48.3, I48.4, I48.91, I48.92) |
| Ventricular arrhythmias, cardiac arrest | 427.1, 427.4x (472.41, 427.42), 427.5, 798, 798.1, 798.2, 798.9, 799.9 | I46.x (I46.2, I46.8, I46.9), I47.2x (I47.20, I47.21, I47.29), I49.0x (I49.01, I 49.02), R99 |
| Cardiac dysrhythmia, unspecified | 427.9 | I49.9 |
| **Venous thromboembolism (VTE)** | | |
| Pulmonary embolism (not septic) | 415.11, 415.13, 415.19 | I26.02, I26.09, I26.92, I26.93, I26.94, I26.99 |
| Deep vein thrombosis (DVTs) of upper/lower extremities and other sites | Upper extremity DVT: 451.83, 451.84, 451.89, 453.82, 453.83, 453.84, 453.85, 453.87  Lower extremity DVT: 451.1x (451.11, 451.19), 451.2, 451.81, 453.4x (453.40, 453.41, 453.42),  Other sites DVT: 451.9, 453.1, 453.2, 453.3, 453.89, 453.9 | Upper extremity DVT: I82.6 (I82.60x, I82.61x, I82.62 x)  Lower extremity DVT: I80.1 (I80.10-180.13), I80.2 (I80.20x, I80.21x, I80.22x, I80.23x, I80.24x, I80.25x, I80.29x), I80.3, I82.4 (I82.40x, I82.41x, I82.42 x, I82.43 x, I82.44 x, I82.45 x, I82.46 x, I82.49 x, I82.4Yx, I82.4Zx)  Other sites DVT: I80.8, I80.9, I82.1, I82.210, I82.220, I82.290, I82.3, I82.A1x, I82.B1x, I82.890, I82.90 |
| Deep phlebothrombosis and thromboembolism in pregnancy and puerperium | 671.3x, 671.4x, 671.5x, 673.2x | O22.3x, O22.5x, O87.1, O88.2x, O871, O873 |
| **Cardiovascular Risk Factors** | | |
| Diabetes mellitus (DM) | 648.0, 250 | O24.0, O24.1, O24.2, O24.3, E10, E11, E12, E13, E14 |
| Gestational DM | 648.8 | O24.4 |
| Preexisting hypertension complicating pregnancy, childbirth, and puerperium (without superimposed preeclampsia); Unspecified maternal hypertension | 401, 402, 403, 404, 405,  642.0, 642.1, 642.2,  642.9x | I10, I11, I12, I13, I15, I16  O10  O16x  Infant code P00.0 |
| Gestational hypertension without significant proteinuria | 642.3 | O13 |
| Preeclampsia, eclampsia | 642.4, 642.5, 642.6, 642.7 | O11, O14.0, O14.1, O14.9, O15 |
| Hyperlipidemia | 272.0, 272.1, 272.2, 272.3, 272.4 | E78.0, E78.1, E78.2, E78.3, E78.4, E78.5 |
| Smoking during pregnancy | 649.0, 305.1 | Z72.0, F17.2 |
| Alcohol use during pregnancy | 303, 305.0 | F10 |
| Drug use during pregnancy | 648.3, 304, 305.2, 305.3, 305.4, 305.5, 305.6, 305.7, 305.8, 305.9 | F11, F12, F13, F14, F15, F16, F18, F19 |
| Depression | 311, 296.2, 296.3 | F32, F33, F34, F38, F39 |
