# Supplementary material for: Racial differences in cardiovascular events and adverse pregnancy outcomes among pregnant individuals with SLE
Source: Lupus Sci Med. 2026 Sep 15;13(2):e001846. doi: 10.1136/lupus-2025-001846 (PMC13583854; doi:10.1136/lupus-2025-001846)
Supplement: Supplementary file 2 [file lupus-13-2-s002.docx]

| **Supplementary Table 2**. ICD-9 and ICD-10 diagnosis and procedure codes and case definitions used to identify non-cardiac Severe Maternal Morbidity (SMM) Indicators | | |
| --- | --- | --- |
|  | **ICD-9 codes** | **ICD-10 codes** |
| Acute renal failure | 584.5, 584.6, 584.7, 584.8, 584.9, 669.3x | N17.x, O90.4 |
| Acute respiratory distress syndrome | 518.5x, 518.81, 518.82, 518.84, 799.1 | J80, J95.1, J95.2, J95.3, J95.82x, J96.0x, J96.2x, J96.9x, R06.03, R09.2 |
| Disseminated intravascular coagulation | 286.6, 286.9, 641.3x, 666.3x | D65, D68.8, D68.9, O45.002, O45.003, O45.009, O45.012, O45.013, O45.019, O45.022, O45.023, O45.029, O45.092, O45.093, O45.099, O46.002, O46.003, O46.009, O46.012, O46.013, O46.019, O46.022, O46.023, O46.029, O46.092, O46.093, O46.099, O67.0, O72.3 |
| Amniotic fluid embolism | 673.1x | O88.112, O88.113, O88.119, O88.12, O88.13 |
| Blood transfusion | 99.0x | 30230H0, 30230K0, 30230L0, 30230M0, 30230N0, 30230P0, 30230R0, 30230T0, 30230H1, 30230K1, 30230L1, 30230M1, 30230N1, 30230P1, 30230R1, 30230T1, 30233H0, 30233K0, 30233L0, 30233M0, 30233N0, 30233P0, 30233R0, 30233T0, 30233H1, 30233K1, 30233L1, 30233M1, 30233N1, 30233P1, 30233R1, 30233T1, 30240H0, 30240K0, 30240L0, 30240M0, 30240N0, 30240P0, 30240R0, 30240T0, 30240H1, 30240K1, 30240L1, 30240M1, 30240N1, 30240P1, 30240R1, 30240T1, 30243H0, 30243K0, 30243L0, 30243M0, 30243N0, 30243P0, 30243R0, 30243T0, 30243H1, 30243K1, 30243L1, 30243M1, 30243N1, 30243P1, 30243R1, 30243T |
| Severe anesthetic complications | 668.0x, 668.1x, 668.2x, 995.4, 995.86 | O29.112–O29.119, O29.122–O29.129, O29.192–O29.199, O29.212–O29.219, O29.292–O29.299, O74.0, O74.1, O74.2, O74.3, O89.0x, O89.1, O89.2, T88.2XXA, T88.3XXA |
| Sepsis | 038.xx, 449, 785.52, 995.91, 995.92, 998.02, 670.2x (after October 1, 2009) | A32.7, A40.x, A41.x, I76, O85, O86.04, R65.20, R65.21, T81.12XA, T81.44XA |
| Hysterectomy | 68.39, 68.49, 68.59, 68.69, 68.79, 68.9 (also include 68.3, 68.4, 68.5, 68.6, 68.7; non-specific codes used frequently) | 0UT90ZL, 0UT90ZZ, 0UT97ZL, 0UT97ZZ |
| Ventilation | 96.70, 96.71, 96.72 | 5A1935Z, 5A1945Z, 5A1955Z |
| Temporary tracheostomy | 31.1 | 0B110F4, 0B113F4, 0B114F4 |
| The ICD-9 and ICD-10 diagnosis and procedure codes for non-cardiac SMM were adapted from the CDC definitions of these SMM indicators  (Source: <https://www.cdc.gov/maternal-infant-health/php/severe-maternal-morbidity/icd.html?CDC_AAref_Val=https://www.cdc.gov/reproductivehealth/maternalinfanthealth/smm/severe-morbidity-ICD.htm>) | | |
