# Supplementary material for: Racial differences in cardiovascular events and adverse pregnancy outcomes among pregnant individuals with SLE
Source: Lupus Sci Med. 2026 Sep 15;13(2):e001846. doi: 10.1136/lupus-2025-001846 (PMC13583854; doi:10.1136/lupus-2025-001846)
Supplement: Supplementary file 3 [file lupus-13-2-s003.docx]

| **Supplementary Table 3. Cardiovascular events (CVEs) and adverse pregnancy outcomes (APOs) compared between racial and ethnic groups in pregnant individuals with SLE, restricted to nulliparous pregnancies (n = 3,147), 2005 – 2020** | | | | | |
| --- | --- | --- | --- | --- | --- |
|  | **Asian** | **Hispanic** | **Non-Hispanic Black** | **Others**** | **Non-Hispanic White** |
| **Nulliparous sample** | **566** | **1,228** | **259** | **142** | **952** |
| **CVE^#^** |  |  |  |  |  |
| **No CVE (n %)** | 552 (97.5) | 1,205 (98.1) | 252 (97.3) | 141 (99.3) | 935 (98.2) |
|  |  |  |  |  | Reference |
| **Any CVE (n %)** | 14 (2.5) | 23 (1.9) | ^a^ | ^a^ | 17 (1.8) |
| RR (95% CI) | 1.4 (0.7, 2.8) | 1.0 (0.6, 2.0) | ^a^ | ^a^ |  |
| aRR (95% CI) | 1.1 (0.5, 2.3) | 0.6 (0.3, 1.1) | ^a^ | ^a^ |  |
| VTE CVE (DVT or PE) (n %) | ^a^ | ^a^ | ^a^ | ^a^ |  |
| RR (95% CI) | ^a^ | ^a^ | ^a^ | ^a^ |  |
| aRR (95% CI) | ^a^ | ^a^ | ^a^ | ^a^ |  |
| Non-VTE CVE (n %) | ^b^ | ^b^ | ^a^ | ^a^ | ^a^ |
| RR (95% CI) | 2.3 (0.9, 5.7) | 1.4 (0.6, 3.4) | ^a^ | ^a^ |  |
| aRR (95% CI) | 1.6 (0.6, 4.3) | 0.7 (0.3, 1.7) | ^a^ | ^a^ |  |
| **Maternal APOs** |  |  |  |  |  |
| **No maternal APO (n %)** | 484 (85.5) | 1,022 (83.2) | 188 (72.6) | 125 (88.0) | 844 (88.7) |
|  |  |  |  |  | Reference |
| **Any maternal APO (n %)** | 82 (14.5) | 206 (16.8) | 71 (27.4) | 17 (12.0) | 108 (11.3) |
| RR (95% CI) | 1.3 (1.0, 1.7) | 1.5 (1.2, 1.9) | 2.4 (1.8, 3.3) | 1.1 (0.6, 1.8) |  |
| aRR (95% CI) | 1.1 (0.8, 1.5) | 1.2 (0.9, 1.5) | 1.9 (1.3, 2.6) | 0.8 (0.5, 1.4) |  |
| Non-cardiac severe maternal morbidity (SMM) (n %) | 23 (4.1) | 50 (4.1) | 15 (5.8) | ^a^ |  |
| RR (95% CI) | 1.7 (0.9, 3.0) | 1.7 (1.0, 2.8)^c^ | 2.4 (1.3, 4.6) | ^a^ |  |
| aRR (95% CI) | 1.1 (0.6, 2.0) | 0.9 (0.5, 1.6) | 1.2 (0.6, 2.4) | ^a^ |  |
| Maternal intensive care use at delivery (n %) | ^a^ | ^a^ | ^a^ | ^a^ | ^a^ |
| RR (95% CI) | ^a^ | ^a^ | ^a^ | ^a^ |  |
| aRR (95% CI) | ^a^ | ^a^ | ^a^ | ^a^ |  |
| Maternal readmission in first year (n %) | 68 (12.0) | 186 (15.2) | 64 (24.7) | 16 (11.3) | 95 (10.0) |
| RR (95% CI) | 1.2 (0.9, 1.6) | 1.5 (1.2, 1.9) | 2.5 (1.8, 3.4) | 1.1 (0.7, 1.9) |  |
| aRR (95% CI) | 1.1 (0.8, 1.6) | 1.2 (0.9, 1.6) | 1.9 (1.3, 2.7) | 0.9 (0.5, 1.5) |  |
| Maternal death in first year (n %) | ^a^ | ^a^ | ^a^ | ^a^ | ^a^ |
| RR (95% CI) | ^a^ | ^a^ | ^a^ | ^a^ |  |
| aRR (95% CI) | ^a^ | ^a^ | ^a^ | ^a^ |  |
| **Infant APOs** |  |  |  |  |  |
| **No infant APO (n %)** | 262 (46.3) | 628 (51.1) | 109 (42.1) | 73 (51.4) | 641 (67.3) |
|  |  |  |  |  | Reference |
| **Any infant APO (n %)** | 304 (53.7) | 600 (48.9) | 150 (57.9) | 311 (32.7) | 69 (48.6) |
| RR (95% CI) | 1.6 (1.4, 1.9) | 1.5 (1.3, 1.7) | 1.8 (1.5, 2.2) | 1.5 (1.1, 1.9) |  |
| aRR (95% CI) | 1.5 (1.3, 1.8) | 1.3 (1.2, 1.6) | 1.6 (1.3, 1.9) | 1.3 (1.0, 1.7)^c^ |  |
| Preterm birth (PTB) (n %) | 139 (24.6) | 291 (23.7) | 80 (30.9) | 26 (18.3) | 120 (12.6) |
| RR (95% CI) | 1.9 (1.5, 2.5) | 1.9 (1.5, 2.3) | 2.5 (1.8, 3.3) | 1.5 (1.0, 2.2) |  |
| aRR (95% CI) | 1.5 (1.2, 2.0) | 1.4 (1.1, 1.7) | 1.9 (1.4, 2.6) | 1.1 (0.7, 1.7) |  |
| *Early PTB (<32 WGA)* (n %) | 33 (5.8) | 81 (6.6) | ^b^ | ^a^ | 21 (2.2) |
| RR (95% CI) | 2.9 (1.7, 5.0) | 3.2 (2.0, 5.2) | 4.4 (2.4, 8.1) | ^a^ |  |
| aRR (95% CI) | 1.6 (0.9, 2.9) | 1.6 (0.9, 2.7) | 2.3 (1.2, 4.5) | ^a^ |  |
| *Moderate PTB (32 - <34 WGA)* (n %) | 19 (3.4) | 41 (3.3) | ^a^ | ^a^ | 12 (1.3) |
| RR (95% CI) | 3.0 (1.5, 6.2) | 2.9 (1.5, 5.6) | ^a^ | ^a^ |  |
| aRR (95% CI) | 1.8 (0.8, 4.0) | 1.8 (0.9, 3.6) | ^a^ | ^a^ |  |
| *Late PTB (34- <37 WGA)* (n %) | 87 (15.4) | 169 (13.8) | 51 (19.7) | 16 (11.3) | 87 (9.1) |
| RR (95% CI) | 1.8 (1.3, 2.4) | 1.6 (1.2, 2.1) | 2.3 (1.7, 3.3) | 1.3 (0.8, 2.2) |  |
| aRR (95% CI) | 1.5 (1.1, 2.1) | 1.3 (1.0, 1.7) | 2.1 (1.4, 3.0) | 1.0 (0.6, 1.8) |  |
| Small-for-gestational age infant (n %) | 156 (27.6) | 255 (20.8) | 60 (23.2) | 25 (17.6) | 136 (14.3) |
| RR (95% CI) | 1.9 (1.5, 2.4) | 1.5 (1.2, 1.8) | 1.6 (1.2, 2.2) | 1.2 (0.8, 1.9) |  |
| aRR (95% CI) | 1.7 (1.4, 2.3) | 1.3 (1.1, 1.7) | 1.5 (1.1, 2.1) | 1.2 (0.7, 1.8) |  |
| Neonatal intensive care use at birth (n %) | 96 (17.0) | 220 (17.9) | 60 (23.2) | 29 (20.4) | 102 (10.7) |
| RR (95% CI) | 1.6 (1.2, 2.1) | 1.7 (1.3, 2.1) | 2.2 (1.6, 3.0) | 1.9 (1.3, 2.9) |  |
| aRR (95% CI) | 1.3 (1.0, 1.7) | 1.2 (1.0, 1.6) | 1.6 (1.1, 2.3) | 1.5 (1.0, 2.4)^c^ |  |
| Infant readmission in the first year (n %) | 58 (10.3) | 155 (12.6) | 32 (12.4) | 14 (9.9) | 80 (8.4) |
| RR (95% CI) | 1.2 (0.9, 1.7) | 1.5 (1.1, 2.0) | 1.4 (1.0, 2.2) | 1.2 (0.7, 2.1) |  |
| aRR (95% CI) | 1.2 (0.8, 1.7) | 1.5 (1.1, 2.0) | 1.3 (0.8, 2.0) | 1.0 (0.6, 1.9) |  |
| Infant death in the first year (n %) | ^a^ | 12 (1.0) | ^a^ | ^a^ | ^a^ |
| RR (95% CI) | ^a^ | 4.7 (1.0, 20.8)^c^ | ^a^ | ^a^ |  |
| aRR (95% CI) | ^a^ | 2.6 (0.5, 12.9) | ^a^ | ^a^ |  |
| Other race includes American Indian/Alaska Native, Native Hawaiian/Pacific Islander, other race, two or more races.  ^a^not displayed and risk not calculated when n < 11; ^b^Value masked to prohibit cell with n<11 calculation; ^c^p <0.05  **Adjusted relative risk (aRR)** adjusted for racial/ethnicity propensity score created from age group, primary payer, maternal education, pre-pregnancy body mass index group, clinical comorbidities (preexisting and gestational diabetes mellitus and hypertension, pregnancy-induced hypertension, hyperlipidemia, depression, alcohol, tobacco, and drug use during pregnancy), presence of concurrent lupus nephritis and presence of antiphospholipid syndrome.  ^#^CVE was defined as a composite of acute myocardial infarction, stroke, heart failure and peripartum cardiomyopathy, myocarditis, pericarditis, dysrhythmia, and venous thromboembolism (pulmonary embolism or deep vein thrombosis) | | | | | |
