# Supplementary material for: Racial differences in cardiovascular events and adverse pregnancy outcomes among pregnant individuals with SLE
Source: Lupus Sci Med. 2026 Sep 15;13(2):e001846. doi: 10.1136/lupus-2025-001846 (PMC13583854; doi:10.1136/lupus-2025-001846)
Supplement: Supplementary file 4 [file lupus-13-2-s004.docx]

| **Supplementary Table 4. Adverse pregnancy outcomes (APOs) among racial/ethnic groups compared to non-Hispanic White individuals with SLE, stratified by CVE status, restricted to nulliparous pregnancies (n = 3,147), 2005 – 2020** | | | | | |
| --- | --- | --- | --- | --- | --- |
|  | **Asian** | **Hispanic** | **Non-Hispanic Black** | **Others**** | **Non-Hispanic White** |
|  |  |  |  |  | **Reference** |
| **SLE without CVE during pregnancy** |  |  |  |  |  |
| **Sample** | **552** | **1,205** | **252** | **141** | **935** |
| **Any maternal APO (n %)** | 75 (13.6) | 194 (16.1) | 67 (26.6) | 17 (12.1) | 105 (11.2) |
| RR (95% CI) | 1.2 (0.9, 1.6) | 1.4 (1.1, 1.8) | 2.4 (1.7, 3.2) | 1.1 (0.6, 1.8) |  |
| aRR (95% CI) | 1.1 (0.8, 1.5) | 1.2 (0.9, 1.5) | 1.8 (1.3, 2.5) | 0.8 (0.5, 1.4) |  |
| **Any infant APO (n %)** | 293 (53.1) | 582 (48.3) | 144 (57.1) | 68 (48.2) | 301 (32.2) |
| RR (95% CI) | 1.6 (1.4, 1.9) | 1.5 (1.3, 1.7) | 1.8 (1.5, 2.2) | 1.5 (1.2, 1.9) |  |
| aRR (95% CI) | 1.5 (1.3, 1.8) | 1.4 (1.2, 1.6) | 1.6 (1.3, 1.9) | 1.3 (1.0, 1.8)^c^ |  |
| ^a^not displayed when n < 11; ^b^not calculated when n < 5 in group or ref  **Comparison group (reference)**: Non-Hispanic White individuals  **aRR** adjusted for racial/ethnicity propensity score created from age group, primary payer, maternal education, pre-pregnancy body mass index group, clinical comorbidities (preexisting and gestational DM and HTN, pregnancy-induced HTN, hyperlipidemia, depression, alcohol, tobacco, and drug use during pregnancy), presence of LN, presence of APS  # aRRs were only estimated for nulliparous SLE pregnancies without a CVE due to low sample size of nulliparous pregnancies with CVE | | | | | |
