# Supplementary material for: Racial differences in cardiovascular events and adverse pregnancy outcomes among pregnant individuals with SLE
Source: Lupus Sci Med. 2026 Sep 15;13(2):e001846. doi: 10.1136/lupus-2025-001846 (PMC13583854; doi:10.1136/lupus-2025-001846)
Supplement: Supplementary file 5 [file lupus-13-2-s005.docx]

| **Supplementary Table 5. Risk of Adverse pregnancy outcomes (APOs) among individuals with SLE with a CVE versus SLE without a CVE, restricted to nulliparous pregnancies, stratified by racial/ethnicity, 2005-2020** | | | | |
| --- | --- | --- | --- | --- |
|  | **Asian** | **Hispanic** | **Non-Hispanic Black** | **Non-Hispanic White** |
| **Any maternal APO** |  |  |  |  |
| RR (95% CI) | 3.7 (1.7, 8.0) | 3.2 (1.8, 5.8) | ^a^ | ^a^ |
| aRR (95% CI) | 2.2 (0.8, 5.6) | 1.6 (0.8, 3.3) | ^a^ | ^a^ |
| Non-cardiac severe maternal morbidity (SMM^#^) |  |  |  |  |
| RR (95% CI) | 11.0 (4.1, 29.5) | 5.8 (2.3, 14.7) | ^a^ | ^a^ |
| aRR (95% CI) | 7.8 (2.3, 26.7) | 2.6 (0.7, 8.8) | ^a^ | ^a^ |
| Acute renal failure |  |  |  |  |
| RR (95% CI) | 38.5 (11.1, 133.0) | ^a^ | ^a^ | ^a^ |
| aRR (95% CI) | 29.3 (7.1, 121.0) | ^a^ | ^a^ | ^a^ |
| Blood transfusion |  |  |  |  |
| RR (95% CI) | ^a^ | ^a^ | ^a^ | ^a^ |
| aRR (95% CI) | ^a^ | ^a^ | ^a^ | ^a^ |
| Maternal readmission in first year |  |  |  |  |
| RR (95% CI) | ^a^ | 2.7 (1.4, 5.2) | ^a^ | ^a^ |
| aRR (95% CI) | ^a^ | 1.1 (0.5, 2.7) | ^a^ | ^a^ |
| **Any infant APO** |  |  |  |  |
| RR (95% CI) | 1.5 (0.8, 2.7) | 1.6 (1.0, 2.6)^b^ | 1.5 (0.7, 3.4) | 1.8 (1.0, 3.4) |
| aRR (95% CI) | 1.1 (0.6, 2.2) | 1.0 (0.6, 1.7) | 1.4 (0.6, 3.2) | 1.6 (0.8, 3.0) |
| Preterm birth (PTB) |  |  |  |  |
| RR (95% CI) | 3.1 (1.6, 5.8) | 1.9 (1.0, 3.5) | 2.4 (1.0, 5.9) | ^a^ |
| aRR (95% CI) | 1.7 (0.8, 3.8) | 0.6 (0.3, 1.2) | 2.2 (0.9, 5.6) | ^a^ |
| Small-for-gestational age infant |  |  |  |  |
| RR (95% CI) | ^a^ | 2.1 (1.1, 4.0) | ^a^ | 2.1 (0.9, 5.1) |
| aRR (95% CI) | ^a^ | 1.4 (0.7, 3.0) | ^a^ | 1.9 (0.8, 4.8) |
| Neonatal intensive care use at birth |  |  |  |  |
| RR (95% CI) | ^a^ | 2.2 (1.1, 4.4) | ^a^ | 2.8 (1.2, 7.0) |
| aRR (95% CI) | ^a^ | 0.8 (0.3, 1.8) | ^a^ | 2.3 (0.9, 5.6) |
| Infant readmission in first year |  |  |  |  |
| RR (95% CI) | ^a^ | ^a^ | ^a^ | ^a^ |
| aRR (95% CI) | ^a^ | ^a^ | ^a^ | ^a^ |
| ^a^Not calculated when n < 11, ^b^p <0.05  Other race includes American Indian/Alaska Native, Native Hawaiian/Pacific Islander, other race, two or more races.  **Comparison group (reference):** **SLE without CVEs within each racial/ethnic group**  **aRR** adjusted for propensity score for CVE created from age group, primary payer, maternal education, pre-pregnancy body mass index group, clinical comorbidities (preexisting and gestational DM and HTN, pregnancy-induced HTN, hyperlipidemia, depression, alcohol, tobacco, and drug use during pregnancy), presence of LN, and presence of APS.  For Others**, the risks were not calculated for APOs due to small sample size  ^#^Non-cardiac components of SMM include acute renal failure, acute respiratory distress syndrome, disseminated intravascular coagulation, amniotic fluid embolism, blood transfusion, severe anesthesia complications, sepsis, hysterectomy, temporary tracheostomy and ventilation | | | | |
